# Asymmetry of Hippocampus and Amygdala Defect in Subjective Cognitive Decline Among the Community Dwelling Chinese

**DOI:** 10.3389/fpsyt.2018.00226

**Published:** 2018-06-11

**Authors:** Ling Yue, Tao Wang, Jingyi Wang, Guanjun Li, Jinghua Wang, Xia Li, Wei Li, Mingxing Hu, Shifu Xiao

**Affiliations:** ^1^Department of Geriatric Psychiatry, Shanghai Mental Health Center, Shanghai Jiao Tong University School of Medicine, Shanghai, China; ^2^Alzheimer's Disease and Related Disorders Center, Shanghai Jiao Tong University, Shanghai, China; ^3^Division of Psychiatry, University of College London, London, United Kingdom; ^4^Department of Computer Science, University of College London, London, United Kingdom

**Keywords:** subjective cognitive decline, Alzheimer's disease, hippocampus, amygdala, asymmetry, mild cognitive impairment

## Abstract

**Background:** Subjective cognitive decline (SCD) may be the first clinical sign of Alzheimer's disease (AD). SCD individuals with normal cognition may already have significant medial temporal lobe atrophy. However, few studies have been devoted to exploring the alteration of left-right asymmetry with hippocampus and amygdala in SCD. The aim of this study was to compare SCD individuals with amnestic mild cognitive impairment (MCI) patients and the normal population for volume and asymmetry of hippocampus, amygdala and temporal horn, and to assess their relationship with cognitive function in elderly population living in China.

**Methods:** 111 SCD, 30 MCI, and 67 healthy controls (HC) underwent a standard T1-weighted MRI, from which the volumes of the hippocampus and amygdala were calculated and compared. Then we evaluated the pattern and extent of asymmetry in hippocampus and amygdala of these samples. Furthermore, we also investigated the relationship between the altered brain regions and cognitive function.

**Results:** Among the three groups, SCD showed more depressive symptoms (*p* < 0.001) and higher percentage of heart disease (16.4% vs. 35.1%, *p* = 0.007) than controls. In terms of brain data, significant differences were found in the volume and asymmetry of both hippocampus and amygdala among the three groups (*P* < 0.05). In logistic analysis controlled by age, gender, education level, depression symptoms, anxiety symptom, somatic disease and lifestyle in terms of smoking, both SCD and MCI individuals showed significant decreased right hippocampal and amygdala volume than controls. For asymmetry pattern, a ladder-shaped difference of left-larger-than-right asymmetry was found in amygdala with MCI>SCD>HC, and an opposite asymmetry of left-less-than-right pattern was found with HC>SCD>MCI in hippocampus. Furthermore, correlation was shown between the volume of right hippocampus and right amygdala with MMSE and MoCA in SCD group.

**Conclusion:** Our results supported that SCD individuals are biologically distinguishable from HC, and this may relate to cognitive impairment, although more longitudinal studies are need to investigate this further.Moreover, different levels of asymmetry in hippocampus and amygdala might be a potential dividing factor to differentiate clinical diagnosis.

## Introduction

Alzheimer's disease (AD), the most prevalent cause of dementia, continues to increase worldwide. The problem is becoming so rampant in China that it‘s estimated the number of Chinese dementia patients will grow rapidly by 314–336% by 2040 (1). Recently, the Chinese Longitudinal Aging Study (CLAS) reported that 4.5% of those older than 60 have AD (2). This is higher than the figure reported by the previous study in China. In order to provide early intervention and delay significant impairment, identification of clinically and cognitively normal individuals who are at risk of AD dementia is paramount, especially in the early stage of the disease.

Research is proving that there is a long preclinical phase of AD during which there is no cognitive dysfunction but AD pathology is already ongoing (3). A few number of studies supported subjective cognitive decline (SCD) may be the first clinical sign of AD even before amnestic mild cognitive impairment (MCI) (4–9). SCD applies to individuals who have self-reported memory-related complaints. Longitudinal studies found that SCD and MCI are associated with a similarly increased risk of AD (8) and with β-amyloid (Aβ) burden, predicting rapid cognitive decline (7, 9). A 4-year follow-up study reported that the risk of developing dementia is doubled in people with SCD compared with those without SCD (10). Strong evidence includes markers of Aβ-amyloid burden in brain (9, 11, 12), in cerebrospinal fluid (CSF) (13, 14), t-tau, p-tau in CSF (15), and two longitudinal cohort studies with autopsies (16, 17), support that SCD is closely related to AD pathology. However, null findings have been reported (18–20) that it may be related to heterogeneity in SCD population. Furthermore, other studies have found that depression is associated with SCD, representing the “false positive” (21, 22). Consequently, a recent review concluded that SCD is unspecific and can be identified in other conditions such as mild vascular brain lesions, frontotemporal dementia or even depression (23). Taken together, SCD may be an early indicator of risk to progress to neurodegeneration, other psychogenic or organic etiologies.

Obviously, SCD alone or in combination with cognitive testing is not sufficient for individual prediction of AD dementia. It has, however, great heuristic value for identification of subjects, which may undergo biomarker-based predementia AD detection. Biomarker-based tests, such as amyloid-PET or CSF studies of amyloid/tau, are good predictors of conversion to dementia, but they are invasive and expensive, and amyloid PET has limited availability (24), which makes it hard to carry out in a large sample. Structural MRI of medial temporal atrophy (MTA) is considered to be the biomarker for an early diagnosis of AD (25) and MCI (26). Furthermore, volume reduction of medial temporal lobe, including hippocampus, amygdala and temporal horn in SCD supports the concept of SCD as a very early manifestation of AD prior to MCI (4–6, 27, 28). Additionally, a study on cognitively intact individuals also showed that preexisting structural volume loss may occur before abnormal amyloid PET (29). Most of the previous structural MRI studies in SCD reported total hippocampus volume loss (5, 6, 30), partially in right hippocampus (7, 31, 32) or right amygdala (6), and recently a study reported small left hippocampus may be associate with depression symptoms in SCD subjects (9).

However, few studies have been devoted to exploring the alteration of left-right asymmetry in individuals of SCD. Previous studies concluded the left-less-than-right pattern in hippocampus was significant in healthy elderly adults, but not in AD (33–35). While in MCI group, the results of asymmetries are inconsistent. A meta-analysis reported an extent of left-less-than-right pattern with MCI>control>AD (35). In contrast, a cohort study reported lesser right asymmetry in MCI compared to controls, especially in hippocampus (36). Though no asymmetry study reported in SCD, a study reported hypometabolism and reduction in the right hippocampus, but not in left hippocampus (7), while another study reported smaller right amygdala in SCD, but not the left (6). These findings suggested a possible laterality effect. As SCD is considered as the earliest stage of AD and based on previous consistent results of the reduced R>L hippocampal asymmetry in AD, it is important to explore the asymmetry of SCD, which may be more sensitive than difference in absolute size and suggestive of which hemisphere or brain region is more vulnerable at the very beginning of the disease.

The CLAS was designed to provide information about the cognitive, mental and psychosocial health of older people in China (37). This survey was a joint effort of 15 institutions located in the eastern, middle, and western parts of China. The sample was randomly selected from all permanent residents aged over 60 in the 2010 national census (37). Our data analysis was carried out in Shanghai (2) and all subjects received MRI scan. No other inclusion or exclusion criteria were applied.

The aim of this study was to compare SCD individuals with normal population, an independent sample from a Chinese Han people community, in terms of volume and asymmetry of hippocampus, amygdala and temporal horn. In addition, individuals with amnestic MCI, which indicated the prodromal stage of AD, from the same community were included. We also assessed the relationship between the changed brain regions with cognitive function in elderly population.

## Materials and methods

### Participants

We report on a subsample from the CLAS study (37), a community-based study of individuals who were all Han people aged 60 and older in Shanghai (2). This study was approved by the Institution's Ethical Committee of Shanghai Mental Health Center, Shanghai Jiao Tong University School of Medicine, and written informed consent was obtained from all subjects and/or their legal guardians. All experimental procedures were carried out in accordance with the approved guidelines and with the principles of the Declaration of Helsinki. A cerebral 3D-Magnetic Resonance Imaging (MRI) sub-study was performed among the participants. Of 1,068 participants, 214 right-handed Han individuals recruited were eligible for the MRI scan in the first wave. Compared with participants who did not have MRI (*n* = 854), those who did were significantly younger (mean age 69.9 ± 7.6 vs. 73.5 ± 8.5) years, *P* < 0.001), and received more years of education (8.5 ± 4.8 vs. 7.4 ± 4.9, *P* = 0.012).

Among the 214 scans performed, six subjects with vascular MCI were excluded from the analysis. In the present study, the sample comprised 30 individuals with amnestic MCI and 178 cognitively normal older subjects including 111 SCD and 67 healthy controls (HC).

### Subjective memory decline

SCD was assessed by self-report. Participants were asked the question “Do you feel you can remember things as well as you used to?” If the answer is “yes,” another question “How long did it last?” was asked. Based on a conceptual framework of criteria for identification of SCD (38), our SCD group should meet the following criteria: (1) the onset age of >60 years old; (2) the presence of gradual memory decline has persisted for ≥6 months; (3) objective memory performance within normal range. Amnestic MCI was classified using the Peterson criteria (39).

### Sociodemographic and health measures

Total years of education, somatic disease (i.e., hypertension, hyperlipidemia, diabetes mellitus, heart disease) and anxiety symptomatology were assessed by self-report. In terms of the anxiety symptom, participants were asked one question “Do you think you tend to feel nervous or anxious?” Depressive symptoms were assessed using a Chinese version of the Geriatric Depression Scale (GDS) (40).

### Neuropsychological tests

All subjects completed a battery of neuropsychological assessments which have been described previously (2). The screening procedures included a Chinese version of the Mini Mental State Examination (MMSE) (41), Montreal Cognitive Assessment (MoCA) (42), WMS-R (43, 44), a Chinese version of the Rey Auditory-Verbal Learning Test (RAVLT) (44), and a Chinese Version of Verbal Associates task (44).

### Magnetic resonance imaging acquisition and processing

All subjects were scanned on a 3.0-tesla MRI scanner (Siemens MAGNETOM VERIO 3.0T, Germen). The parameters of T1-weighted 3D magnetization prepared rapid gradient echo (MPRAGE) sequences were as follows: TR = 2,300 ms, TE = 2.98 ms, flip angle of 9 degree; matrix size = 240 × 256; field of view (FOV) = 240 × 256 mm; slice thickness = 1.2 mm.

Automated procedures were used to ascertain volumetric data. The automated assessment was described using the Learning Embedding for Atlas Propagation (LEAP) algorithm (45). For each subject, volume and asymmetry with hippocampus, amygdala and temporal horn as well as the brain size index were extracted. Collected datasets were analyzed without knowing the cognitive state or other clinical data about the subjects. In addition, to assess the role of differences in left and right, an asymmetry index was computed using the equation: [right volume-left volume]/[total volume] × 100%.

### Statistical analyses

All analyses were performed using the Statistical Package for Social Sciences (SPSS 19.0). Group differences in demographic data were assessed using a one-way independent ANOVA. Bonferroni corrected *post-hoc* tests were conducted for between group comparisons of continuous data (0.05/3 = 0.017). If the homogeneity of variance was violated, the Kruskal-Wallis tests were used as well as for pairwise comparison. Pearson's chi-square tests were conducted for categorical variables. Variables of age, gender, years of education and GDS score were significantly different among groups, hence, ANCOVA was built with the neuropsychological scores as dependent variable, gender and group as fixed factors, and age, years of education and GDS score as covariates.

In addition, a stepwise approach was used the MRI data evaluation. In the first step, in order to find the candidate brain region, the brain data were assessed in a univariate analysis while the brain size index was adjusted to control the individual difference. After that, the regional brain variables were further analyzed using binary logistic regression in two models. We treated the group as dependent variable, and in Model 1 using gender, age, years of education and brain size index as covariates. As previous literature confirmed that a few major factors accounting for AD include depression, hypertension, diabetes, physical inactivity, smoking (46), so in Model 2, we additionally adjusted for GDS score, self-report anxiety, hypertension, hyperlipidemia, diabetes mellitus, Heart disease and smoking as covariates. Contrasts were calculated by SCD vs. HC, MCI vs. HC, and SCD vs. MCI, respectively. Pearson's correlative analysis was performed to examine relationships between structural data and neuropsychological performances using age as covariate.

## Results

### Demographic and neuropsychological testing

Table 1 presents the demographic and neuropsychological characteristics. Compared with HC group, SCD group showed higher GDS score, indicating more depression symptoms (*p* < 0.001) and higher percentage of heart disease (16.4% vs. 35.1%, *p* = 0.007). Age, gender, years of education, anxiety symptom distribution did not differ significantly, although SCD group had slightly fewer male subjects than HC group (45% vs. 59.7%, *p* = 0.07).

**Table 1:** Demography and neuropsychological test among SCD, MCI and HC.

	**SCD (*n* = 111)**	**MCI (*n* = 30)**	**HC (*n* = 67)**	**F/λ^2/^H**	***Post-hoc***
Age (year)	69.8 ± 7.6	75.5 ± 7.6	67.7 ± 6.6	11.80**	HC, SCD < MCI*
Male, n (%)	50(45.0%)	8(26.7%)	40(59.7%)	9.49*	HC, SCD*>MCI*
Education (year)	9.0 ± 4.1	3.9 ± 4.5	9.2 ± 4.2	19.74**	HC, SCD < MCI**
GDS	3.5 ± 4.1	5.5 ± 5.7	1.8 ± 3.8	30.78**^#^	HC < MCI**^#^; HC < SCD**^#^
Self-report anxiety, n (%)	5(4.5%)	1(3.3%)	0(0%)	n.s.	NS
Smoke^a^	35(31.5%)	4(13.3%)	22(32.8%)	n.s.	NS
MMSE	27.3 ± 2.3	20.2 ± 4.4	28.1 ± 1.7	70.31**	HC,SCD>MCI**
MoCA	24.0 ± 4.2	13.7 ± 4.4	25.3 ± 3.5	52.08**	HC,SCD>MCI**
Hypertension, n (%)	58(52.3%)	12(40%)	27(40.3%)	n.s.	NS
Hyperlipidemia, n (%)	22(19.8%)	6(20.0%)	6(9.0%)	n.s.	NS
Diabetes mellitus, n (%)	23(20.7%)	6(20.0%)	7(10.4%)	n.s.	NS
Heart disease, n (%)	39(35.1%)	8(26.7%)	11(16.4%)	7.31*	SCD>HC*
Digit span forward^a^	8.87 ± 2.47	5.54 ± 1.92	9.93 ± 2.25	35.94**	HC,SCD>MCI**; HC>SCD*
Digit span backward^b^	5.48 ± 2.07	3.17 ± 1.97	6.20 ± 2.38	19.98**	HC,SCD>MCI**
Auditory verbal learning (AVLT)	31.34 ± 9.02	20.27 ± 7.73	34.34 ± 10.12	24.54**	HC,SCD>MCI**
Associative learning^b^	6.54 ± 3.16	2.24 ± 2.02	7.20 ± 3.37	33.41**	HC,SCD>MCI**
Verbal fluency^c^	27.51 ± 8.39	17.90 ± 7.78	30.50 ± 10.33	20.02**	HC,SCD>MCI**
Visual recognition (functional)^b^	3.43 ± 0.80	2.43 ± 0.86	3.77 ± 0.46	34.34**	HC,SCD>MCI**, HC>SCD*
Visual recognition (semantic) ^b^	3.04 ± 1.21	2.03 ± 1.03	3.47 ± 0.71	18.88**	HC,SCD>MCI**
Visual matching and reasoning^b^	5.40 ± 2.34	3.60 ± 1.58	6.13 ± 2.12	16.84**	HC,SCD>MCI**
Visual recognition correct^b^	5.83 ± 1.60	5.37 ± 1.73	6.39 ± 1.20	5.12*	HC>MCI*
WAIS picture completion	10.22 ± 3.81	6.87 ± 4.51	11.97 ± 4.10	16.60**	HC,SCD>MCI**. HC>SCD*
WAIS block design^c^	27.39 ± 8.63	16.33 ± 8.39	29.64 ± 7.15	28.67**	HC,SCD>MCI**

Continuous data presented as mean ± standard deviation; Post-hoc group comparisons-Bonferroni corrected (0.05/3 = 0.017); All the analyses of neuropsychological variables were conducted with age, gender, education and GDS score as covariates. Symbol: NS denotes non-significant; ^#^Kruskal-Wallis test was used. *p < 0.05; **p < 0.001.

a One sample data were missing.

b Two sample data were missing.

c Three sample data were missing.

MCI group showed older age, more females, fewer years of education compared with HC and SCD group. Moreover, MCI group showed higher GDS score than HC group (*p* < 0.001), but not statistically different from SCD group. The proportion of self-report anxiety, hypertension, hyperlipidemia, diabetes mellitus and the lifestyle smoking did not differ between the three groups.

In terms of neuropsychological tests, MCI group exhibited cognitive function deficit in every measurement compared to other two groups. Between HC and SCD, SCD group received significant lower scores than HC group on digit span forward (8.87 ± 2.47 vs. 9.93 ± 2.25), functional visual recognition (3.43 ± 0.80 vs. 3.77 ± 0.46) and WAIS picture completion (10.22 ± 3.81 vs. 11.97 ± 4.10). All the comparisons of diagnostic groups for neuropsychological variables were controlled for age, gender, education and GDS score.

### Medial temporal volume

In univariate ANOVA analysis (Table 2), significant differences were found in the volume and asymmetry of both hippocampus and amygdala between three groups (*p* < 0.017), while the brain size index and temporal horn did not differ between the three groups (*p* > 0.1).

**Table 2:** Medial temporal volumetric measures of the SCD, MCI and HC groups.

	**SCD**	**MCI**	**Control**	***F***	**ANCOVA**		
	**(*n* = 111)**	**(*n* = 30)**	**(*n* = 67)**		**HC vs. SCD**	**HC vs. MCI**	**SCD vs. MCI**
Brain size index	0.80 ± 0.09	0.80 ± 0.07	0.81 ± 0.08			n.s.	
**HIPPOCAMPUS**							
Left	2.33 ± 0.86	2.18 ± 0.28	2.42 ± 0.27	8.44**	NS	HC>MCI**	NS
Right	2.52 ± 0.32	2.28 ± 0.34	2.66 ± 0.29	17.78**	HC>SCD*	HC>MCI**	SCD>MCI*
Asymmetry (%)	3.98 ± 3.31	2.19 ± 4.07	5.08 ± 4.31	6.076*	NS	HC>MCI*	NS
**AMYGDALA**							
Left	1.74 ± 0.24	1.62 ± 0.20	1.80 ± 0.25	5.92*	NS	HC>MCI*	NS
Right	1.47 ± 0.20	1.32 ± 0.23*	1.56 ± 0.20	16.91**	HC>SCD*	HC>MCI**	SCD>MCI*
Asymmetry (%)	−8.41 ± 5.98	−10.49 ± 5.75*	−6.88 ± 4.11	4.785*	NS	HC>MCI*	NS
**TEMPORAL HORN**							
Left	0.69 ± 0.14	0.73 ± 0.27	0.69 ± 0.13			NS^#^
Right	0.55 ± 0.16	0.62 ± 0.24	0.53 ± 0.12			NS^#^	
Asymmetry (%)	−11.78 ± 11.19	−10.04 ± 10.27	−13.20 ± 10.06	NS

Asymmetry is defined as [right volume-left volume]/[total volume]*100%; Post-hoc was Bonferroni corrected (p < 0.05/3 = 0.017); the regional MRI data were adjusted for brain size index; ^#^Kruskal-Wallis test was used (unadjusted). Symbol: NS denotes non-significant.

**Figure 1:**
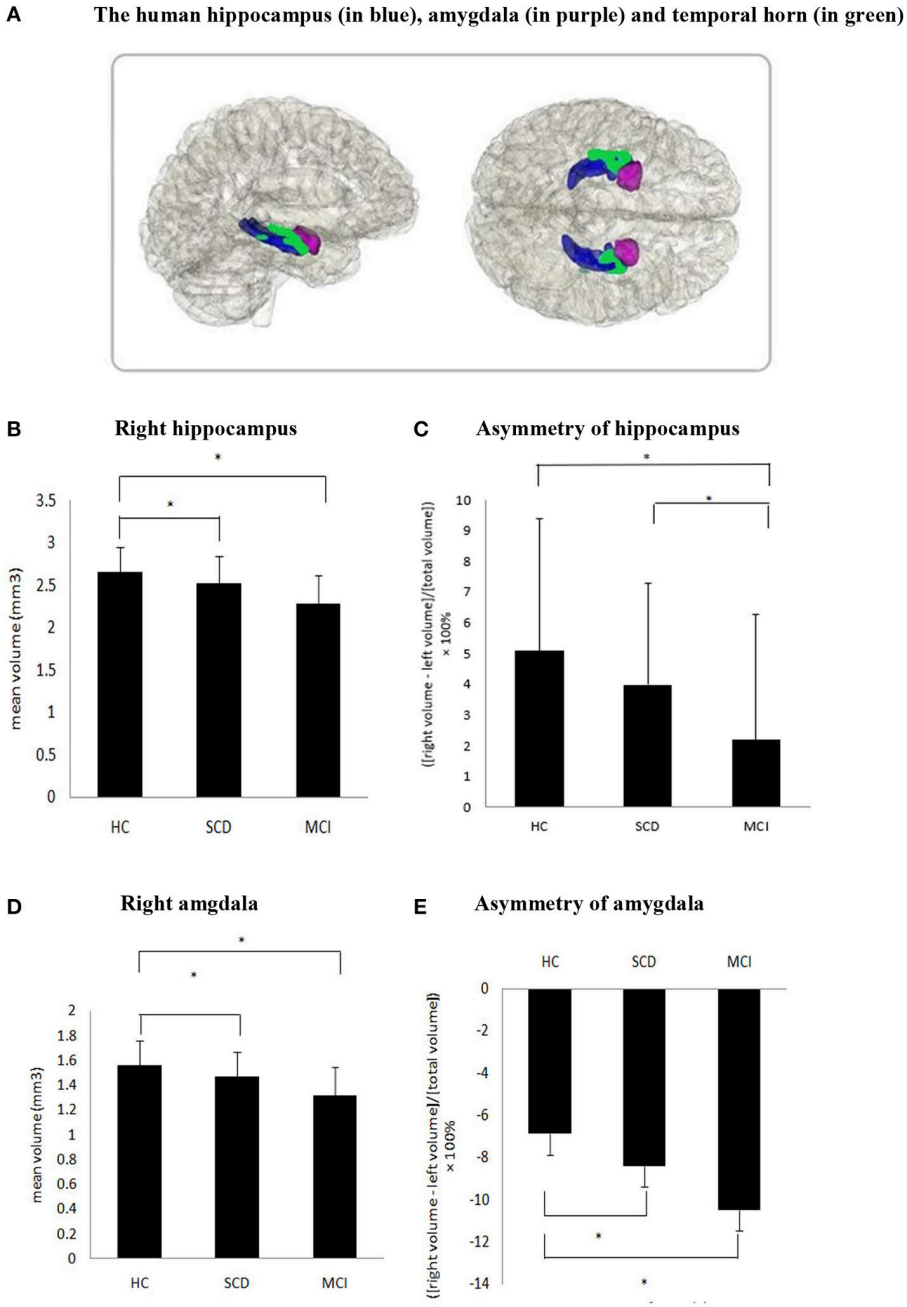
**(A)** The human hippocampus (in blue), amygdala (in purple) and temporal horn (in green). **(B,D,E)** Significant difference was found in the volume of the right hippocampus, right amygdala and asymmetry of amygdala in SCD compared to HC as well as MCI compared with HC. **(C)** Asymmetry of hippocampus between SCD and HC did not reach statistical significance (*P* = 0.07), but significant difference were found in MCI vs. HC and SCD vs. MCI.

In order to address the possible confounding effect, logistic regression analysis was used to assess the association between structural variables and different diagnosis groups in two models (Table 3 and Figure 1). Between SCD and HC group, significant differences were found in the volume of the right hippocampus (odds ratios (OR) for model 1: 0.15, *p* = 0.005, and model 2: 0.09, *p* = 0.001), right amygdala (OR for model 1: 0.07, *p* = 0.012, and model 2: 0.04, *p* = 0.005), and asymmetry of amygdala (OR for model 1: 0.93, *p* = 0.035, and model 2: 0.93, *p* = 0.035), while the asymmetry of hippocampus showed a tendency difference in both models (*p* = 0.07).As expected, HC and MCI group also showed similar differences in the two models. Between SCD and MCI, significant difference was found in asymmetry of hippocampus in Model 1 only (OR: 1.16, *p* = 0.037), and a trend difference in asymmetry of amygdala (*p* = 0.051). Additionally, no significant difference was found between SCD and MCI in the regional brain volumes. No group difference was found in left hippocampus and amygdala volume after adjustment.

**Table 3:** Logistic regression analysis among SCD, MCI and controls after multivariable adjustment.

	**SCD vs. HC**				**MCI vs. HC**				**SCD vs. MCI**			
	**Model 1**		**Model 2**		**Model 1**		**Model 2**		**Model 1**		**Model 2**
	**OR (95%CI)**	***p***	**OR (95%CI)**	***p***	**OR (95%CI)**	***p***	**OR (95%CI)**	***p***	**OR (95%CI)**	***p***	**OR (95%CI)**	***p***
Right hippocampus	**0.15(0.38–0.56)**	**0.005**	**0.09(0.02–0.39)**	**0.001**	**0.35(0.00–0.57)**	**0.019**	**0.02(0.00–0.98)**	**0.049**	0.46(0.08**–**2.64)	0.386	0.73(0.10–5.37)	0.756
Asymmetry of hippocampus	0.92(0.84–1.01)	0.07	0.92(0.84–1.01)	0.07	**0.79(0.66–0.95)**	**0.012**	**0.75(0.58–0.97)**	**0.030**	**0.86(0.75–0.99)**	**0.037**	0.88(0.75–1.02)	0.087
Right amygdala	**0.07(0.01–0.55)**	**0.012**	**0.04(0.01–0.38)**	**0.005**	**0.01(0.00–0.72)**	**0.035**	0.004(0.00–1.12)	0.055	0.19(0.01–3.55)	0.265	0.66(0.02–19.15)	0.809
Asymmetry of amygdala	**0.93(0.88–1.00)**	**0.035**	**0.93(0.87–0.99)**	**0.035**	**0.84(0.73–0.96)**	**0.009**	**0.77(0.63–0.93)**	**0.008**	0.92(0.85–1.00)	0.051	0.94(0.86–1.02)	0.146

*Significant coefficients and p-values are in bold font (p < 0.05). Model 1 was adjusted for gender, age, years of education and brain size index; Model 2 was additionally adjusted for GDS score, self-reported anxiety, hypertension, hyperlipidemia, diabetes mellitus, heart disease and lifestyle of smoking*.

### Correlation analysis of significant brain differences and neuropsychological tests

In a correlation analysis in SCD individuals, we found a significant association between the volume of right amygdala with MMSE (*r* = 0.281, *p* = 0.003) and MoCA (*r* = 0.246, *p* = 0.01), and a correlation between the volume of right hippocampus with MMSE (*r* = 0.243, *p* = 0.011) and MoCA (*r* = 0.208, *p* = 0.03). There was no association between any of the other cognitive functions and these two brain regions. In MCI group, significant associations were found between volume of right hippocampus with MMSE (*r* = 0.433, *p* = 0.024) and MoCA (*r* = 0.398, *p* = 0.040). No other significant results were observed after corrected for age.

## Discussion

Across all individuals, we observed volume of different regions in medial temporal lobe in a sample of community-dwelling population in China. The main finding in our study showed that right hippocampus and right amygdala with significant atrophy in MCI, followed by SCD, compared to controls. We also observed different degrees of amygdala and/or hippocampal asymmetry among the three groups. The differences between SCD and controls remained with no substantial diminution in strength after adjustment by chronic disease, life style, depression or anxiety. This result supported the concept of SCD as a risk factor of degeneration progress and the asymmetry in amygdala and hippocampus may be a more sensitive biomarker than the absolute volume.

### Neuropsychological manifestation in SCD

Our SCD subjects performed slightly poorer in some neuropsychological tests than the controls, even after controlled for age, gender, education and GDS score. This slightly poorer performance in SCD is consistent with previous study (47). Though SCD exhibited a normal global cognition manifested in similar MMSE and MoCA, our result may indicate a risk of future cognitive decline, which is proposed in current concepts that SCD is an important component of the preclinical AD-dementia trajectory (48). In our study, SCD gained lower scores in three tests, named digit span forward, functional visual recognition and WAIS picture completion. While the digit Span Forward test measures attention and concentration (43), functional visual recognition test (49) relates to visual memory and WAIS picture completion test (43) assesses spatial memory, our result revealed that the slight cognitive deficit in SCD covered different cognition domains. This style of cognitive performance was consistent with the idea that SCD was non-specific (23), and could be affected by several conditions, such as Cerebral small vessel disease (CSVD) (50, 51), depression (22) and frontotemporal dementia (52). Consequently, more sensitive neuropsychological tests and repeating these tests over time are needed to identify the root cause of cognitive decline and clarify which neuropsychological test could be the best predictor for the transition from the preclinical to the prodromal stage of the disease.

### Depression and somatic disease in SCD

Depression and anxiety are both important factors that are known to be associated with cognitive decline. In our study, SCD subjects showed more severe depressive symptoms than the control group. The result was consistent with previous studies (4, 9, 53), which reported greater depressive symptomatology in SCD. As mood disorder, especially late-onset depression, in the elderly may be an early manifestation due to AD (54, 55), it is hard to interpret whether the core affective symptoms influence the subjective cognition function assessment. Though our SCD subjects scored slightly higher on the depression scale without being clinically diagnosed with depression, early intervention is recommended on both cognition and emotion for individuals of SCD.

Several somatic and lifestyle factors may contribute to cognitive deficit (46). In our study, we adjusted for related factors in multivariable logistic analysis, including GDS score, self-report anxiety, hypertension, hyperlipidemia, diabetes mellitus, heart disease and lifestyle of smoking. Our SCD samples reported more heart diseases than controls. The result was comparable with a large sample study which reported that cardiovascular disease were associated with considerable cognitive impairment (56). The author also reported that the putative risk factor for cognitive decline was vascular disease due to atherosis. As MCI have been considered possibly at a risk of developing dementia phenotypes other than AD, such as vascular dementia (57), the pre-MCI stage, SCD subjects also under the risk of converting to vascular MCI. Another study which reported that the significant association between SCD and increase in white matter lesions also attributed to the vascular origin (50). What's more, depression and anxiety also impact the heart, therefore, it is still not clear whether the association of SCD with heart disease is synchronous due to vascular lesion or causal interaction with affective symptom. Importantly, elderly people who have heart disease and reported memory decline need a better management of vascular factors.

### Volumetric alteration in hippocampus and amygdala

We found smaller right hippocampus and right amygdala in SCD and MCI compared to HC, and indeed, cognitively normal individuals with SCD showed similar medial temporal lobe structures compared with MCI after controlling for relevant variables. These findings provided more evidence that SCD has biologically changed in brain structure and may be at pre-MCI stage. Our results are consistent with a MRI study which reported SCD and amnestic MCI showed similar patterns of decreased gray matter relative to HC on whole-brain analysis including medial temporal region (28). Several longitudinal studies have verified that the volume atrophy preceded cognitive decline. A 6-year follow-up study including 511 elderly individuals showed that atrophy of the hippocampus and amygdala on MRI in cognitively intact elderly people predicted dementia (30). Another 10 years study demonstrated that right hippocampus changes occurred years before clinical cognitive decline in AD (31). Recently, an 18 month follow-up study divided the 455 healthy elderly subjects into stable controls and deteriorating controls, the result showed that decreased hippocampal and right amygdala volumes preceded the first signs of cognitive decline (58). Our result was consistent with the above research, suggesting that SCD individuals with normal cognition assessment result may already have had significant atrophy in hippocampus and amygdala, mostly in the right hemisphere. Follow-up observation of the current sample may help to interpret the relationship between the brain change and cognitive function in the future.

### Asymmetry change in hippocampus and amygdala

It is interesting to mention the novel findings for the different degrees of asymmetry from both hippocampus and amygdala among MCI, SCD and HC. Our results showed an accordant sequence, the degree of asymmetry with L>R in amygdala was MCI>SCD>HC, and by contrast the asymmetry with R>L in hippocampus was HC>SCD>MCI (see Figure 1). Combined with our result of significantly decreased right hippocampus and right amygdala, the trajectory was going in the same direction. These findings suggest that larger left amygdala asymmetry and less right hippocampal asymmetry may be associated with cognitive decline and mainly co-occur with right medial temporal lobe atrophy. Our result was consistent with previous studies in normal elderly people and AD patients. In HC, previous study reported that the hippocampus has a left-less-than-right asymmetrical structure (59) and another study of meta analysis concluded the asymmetry in amygdala is consistently slightly larger in the left side, though not significant (60). Meanwhile, longitudinal studies in AD have consistently reported the laterality of hippocampus changes to be non-significant when converting to dementia. A study reported the right hippocampus was significantly greater than the left in Clinical Dementia Rating (CDR) 0 (normal) and CDR 0.5 (questionable cognition), but not in CDR 1(mild dementia) (33). Furthermore, a longitudinal study reported a left-less-than-right pattern of hippocampus in the baseline of AD patients, but this result was not repeated at a follow-up scan conducted 15 months later (34).The above study suggested the hippocampal asymmetry in AD was reduced with disease progression and reflected in greater atrophy in the right than the left. Moreover, the reduction in right hippocampus and amygdala in SCD is repeatedly reported (31, 32, 58, 61). A FDG-PET and MRI study found that SCD were associated with smaller right and with hypermetabolism in the right hippocampus, with no significant differences in the corresponding left structures, thus suggestive of a possible laterality effect (7). Though the opposite results that changes in the left hemisphere happened earlier and progress faster were also reported (36). Taken together, our ladder-like progress asymmetry in hippocampus and amygdala among three different levels of cognition group showed that cognitive decline may disrupt the normal asymmetry of hippocampus and amygdala. Comparing our absolute MRI result of hippocampus and amygdala with the relative data of their asymmetry, the alteration in asymmetry may be a more sensitive and earlier assessment index which should be verified by tracing the prognosis of the participants.

### Correlation between brain data and neuropsychological tests

In our study, both the right hippocampus and right amygdala was found associated with MMSE and MoCA. As both MMSE and MoCA are assessed for global cognition, our result is consistent with the common knowledge that both of the two regions are important for cognition. Other study reported SCD with smaller left hippocampal volume was associated with greater depressive symptomatology (9). The left and right hemispheres exhibit functional differences, for instance, the left is more dominant for verbal cognitive function, and the right hemisphere is the dominant hemisphere for the spatial cognitive function (62). Our data did not find the association between the regional brain volume and the specific neuropsychological measurement, probably because SCD is a very early stage. Additionally, it is necessary to mention that recently a hippocampal function-based test called 4 Mountain test which used to test spatial memory showed high sensitivity and specific for MCI due to AD (63) and the preclinical AD (64). Future studies need to confirm whether the right side is the first impaired hemisphere.

### Limitation

This study has several limitations. Firstly, the cross-sectional study is not able to examine causal relationships between specific neuroimaging changes and individual cognitive decline. Follow-ups are needed to make the final diagnosis for these participants. Furthermore, we did not include FDG-PET, amyloid markers and APOE genotype in this work so that the real extent of AD pathology remains unknown. Moreover, recently a new method for shape-based asymmetry analysis of dementia reported more predictive of Alzheimer's disease than volume asymmetry (65). Future studies may use the novel technique in SCD group to compare the volume and shape asymmetry, as well as different hippocampal and amygdala subregions. In addition, recent study (66) also reported smaller brain volumes in adults with hearing loss and an association between hearing loss and cognitive impairment. Further research, especially a follow-up study is needed to clarify this issue.

## Conclusions

Our study reported reduction in right hippocampus and right amygdala in SCD compared to controls and found a ladder-shaped difference of left-larger-than-right asymmetry in amygdala with MCI>SCD>HC, and an opposite asymmetry of left-less-than-right pattern in hippocampus with HC>SCD>MCI. Our results supported that SCD is biologically distinguishable from HC, and that SCD may be the earliest stage of neurocognitive disorder. Assessment of the asymmetry in amygdala and hippocampus may be a particularly sensitive indicator at detecting the earliest cognitive deficits.

## Author contributions

LY, TW, and SX designed the study. MH and SX were responsible for the study, supervised data collection, interpreted the data and offered significant comments on the manuscript. LY, TW, JyW, and WL analyzed the data and wrote the first draft of the manuscript. GL, JhW, and XL provided clinical diagnosis and ratings and offered significant comments on the manuscript. All authors reviewed the manuscript.

### Conflict of interest statement

The authors declare that the research was conducted in the absence of any commercial or financial relationships that could be construed as a potential conflict of interest.
